# Endoscopic transorbital approach to the cavernous sinus: Cadaveric anatomy study and clinical application (^‡^SevEN-009)

**DOI:** 10.3389/fonc.2022.962598

**Published:** 2022-08-26

**Authors:** In-Ho Jung, Jihwan Yoo, Seonah Choi, Seung Hoon Lim, JaeSang Ko, Tae Hoon Roh, Je Beom Hong, Eui Hyun Kim

**Affiliations:** ^1^ Department of Neurosurgery, Yonsei University College of Medicine, Seoul, South Korea; ^2^ Department of Neurosurgery, Dankook University College of Medicine, Cheonan, South Korea; ^3^ Brain Tumor Center, Gangnam Severance Hospital, Seoul, South Korea; ^4^ Department of Neurosurgery, College of Medicine, Kyung Hee University, Seoul, South Korea; ^5^ Department of Ophthalmology, Severance Hospital, Institute of Vision Research, Yonsei University College of Medicine, Seoul, South Korea; ^6^ Endoscopic Skull Base Center, Severance Hospital, Seoul, South Korea; ^7^ Department of Neurosurgery, Ajou University Hospital, Ajou University School of Medicine, Suwon, South Korea; ^8^ Kangbuk Samsung Hospital, Sungkyunkwan University School of Medicine, Seoul, South Korea

**Keywords:** cavernous sinus, endoscope, lateral compartment, skull base tumor, transorbital

## Abstract

**Objective:**

Cavernous sinus (CS) invasion is frequently encountered in the management of skull base tumors. Surgical treatment of tumors in the CS is technically demanding, and selection of an optimal surgical approach is critical for maximal tumor removal and patient safety. We aimed to evaluate the feasibility of an endoscopic transorbital approach (ETOA) to the CS based on a cadaveric study.

**Methods:**

Five cadaveric heads were used for dissection under the ETOA in the comparison with the endoscopic endonasal approach (EEA) and the microscopic transcranial approach (TCA). The CS was exposed, accessed, and explored, first using the ETOA, followed by the EEA and TCA. A dedicated endoscopic system aided by neuronavigation guidance was used for the procedures. During the ETOA, neurovascular structures inside the CS were approached through different surgical triangles.

**Results:**

After completing the ETOA with interdural dissection, the lateral wall of the CS was fully exposed. The lateral and posterior compartments of the CS, of which accessibility is greatly limited under the EEA, were effectively approached and explored under the ETOA. The anteromedial triangle was the largest window *via* which most of the lateral compartment was freely approached. The internal carotid artery and abducens nerve were also observed through the anteromedial triangle and just behind V1. During the ETOA, the approaching view through the supratrochlear and infratrochlear triangles was more directed towards the posterior compartment. After validation of the feasibility and safety based on the cadaveric study, ETOA was successfully performed in a patient with a pituitary adenoma with extensive CS invasion.

**Conclusions:**

Based on the cadaveric study, we demonstrated that the lateral CS wall was reliably accessed under the ETOA. The lateral and posterior compartments of the CS were effectively explored *via* surgical triangles under the ETOA. ETOA provides a unique and valuable surgical route to the CS with a promising synergy when used with EEA and TCA. Our experience with a clinical case convinces us of the efficacy of the ETOA during surgical management of skull base tumors with CS-invasion.

## Introduction

The cavernous sinus (CS) is a venous sinus deep-seated in the central skull base. Radical surgical resection is not generally feasible because CS exploration involves potential risks of complications such as massive venous bleeding, cranial neuropathy, and internal carotid artery (ICA) injury. However, cavernous sinus exploration is often necessary, especially during treatment of various skull base tumors such as endocrine-active pituitary adenomas, chordomas, meningiomas and, at the least, for debulking non-functioning adenomas. Surgeons should be prepared to have a comprehensive anatomical understanding and delicate surgical technique when they access the CS (1–4).

The transcranial approach (TCA) has been a mainstay of cavernous sinus exploration for various conditions such aneurysms, meningiomas, and pituitary adenomas (5–7). The TCA is basically a lateral-to-medial approach and intracavernous neurovascular structures are accessed through several windows between each cranial nerve in the lateral CS wall. Therefore, a certain degree of cranial nerve violation is inevitable. Also, access to the medial compartment of the CS is limited (2, 3). Extensive loss of bony structure, excessive brain retraction, and long operation times are additional disadvantages of the TCA.

**Figure 1:**
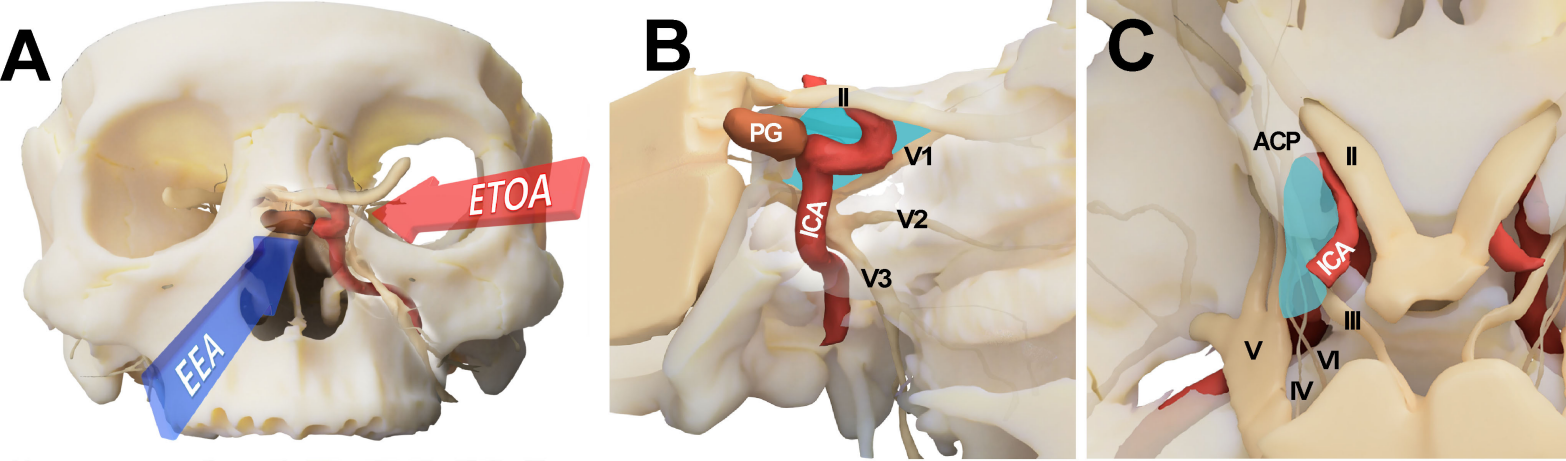
Conceptual illustration for endoscopic transorbital approach (ETOA) and endoscopic endonasal approach (EEA) to the cavernous sinus. **(A)** The cavernous sinus was approached from the infero-medial side under EEA whereas ETOA accesses cavernous sinus from the superolateral side. **(B, C)** Consequently, the lateral compartment of cavernous sinus (blue shadow) was more directly accessed under ETOA while its access was hindered by the tortuous course of internal carotid artery under EEA. ACP, anterior clinoid process; ICA, internal carotid artery; II, optic nerve; III, oculomotor nerve; IV, trochlear nerve; PG, pituitary gland; V, trigeminal nerve; V1, ophthalmic branch of trigeminal nerve; V2, maxillary branch of trigeminal nerve; V3, mandibular branch of trigeminal nerve; VI, abducens nerve.

The endoscopic endonasal approach (EEA) is another major surgical corridor to the CS. Especially during surgery for pituitary adenomas, where the EEA is the choice of surgical corridor in most cases, CS can be explored with various modifications (8, 9). The transnasal corridor is basically an anteromedial-to-posterolateral approach. Because neurovascular structures are less crowded in the medial compartment, surgical morbidity is lower for the EEA than for the TCA. However, access to the lateral compartment is greatly limited by tortuous course of the ICA (**Figure 1**) (8, 10–12).

Recently, endoscopic transorbital approach (ETOA) has been proposed as an alternative route to access various skull base regions. Especially, it has been proved that the medial temporal lobe is effectively approached by ETOA in many studies, which suggests it is a promising surgical corridor to access the CS (4). However, the potential benefits of CS exploration using the ETOA have not been fully investigated. We postulated that the ETOA is an effective alternative to access the lateral compartment of the CS. We aimed to evaluate the feasibility of the ETOA for access to the CS, compared with the TCA and EEA.

## Materials and methods

Five human cadaveric heads provided by the Surgical Anatomy Education Center of Yonsei University College of Medicine were used for this study. Three cadaveric heads were prepared using Thiel embalming and ethanol-glycerin fixation, and two were fresh cadavers. All cadaveric heads were injected with silicone rubber compounds (MICROFIL^®^; Flow Tech, Inc., Carver, MA, USA) to fill and opacify the vessels. Before starting dissection, the cadaveric heads were examined using computed tomography (CT) scans to obtain images that were used by the neuro-navigation system (Stryker navigation system, Kalamazoo, MI) during dissection. For cadaveric dissection under ETOA and EEA, a rigid endoscope 4 mm in diameter and 18 cm in length with 0° and 30° optic lenses was used (Stryker neuroendoscopy, Kalamazoo, MI, and Arthrex Synergy UHD4 endoscopy, Naples, FL). During the TCA, a conventional skull base approach was performed, aided by a surgical microscope (Carl Zeiss, Oberkochen, Germany) with a 2D medical camera system (3D Medivision, Seoul, Republic of Korea). The cadaveric study committee of the Yonsei University of Medicine and the Institutional Review Board of Severance Hospital, Yonsei University College of Medicine approved this study. After testing feasibility and safety based on this cadaveric study, combined EEA and ETOA was performed to treat a patient with a recurrent invasive pituitary adenoma.

### Endoscopic transorbital approach

**Figure 2:**
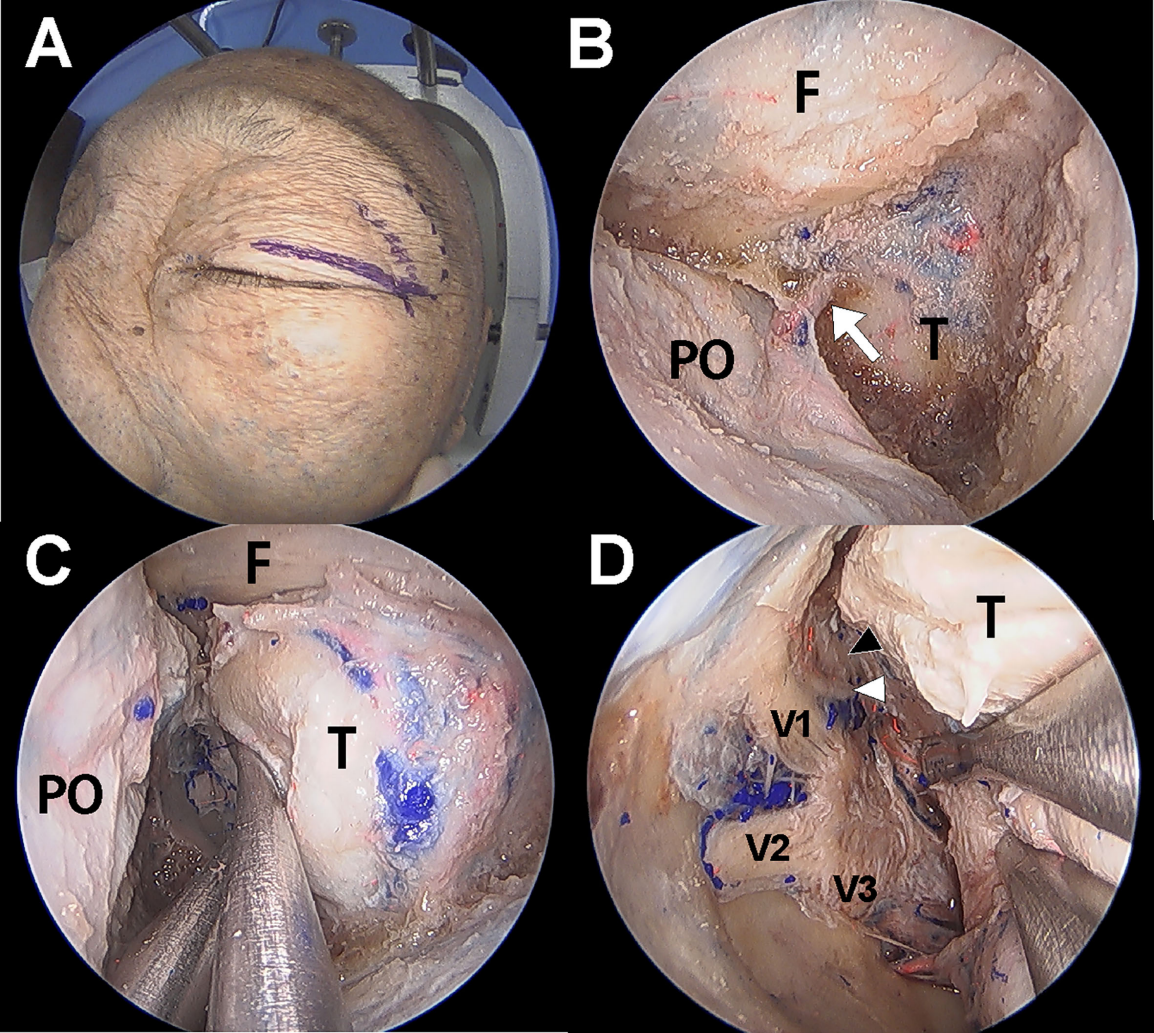
Cadaver demonstration showing the endoscopic transorbital approach (ETOA) to cavernous sinus (CS). **(A)** Superior eyelid incision was made on the superior eyelid with lateral extension. **(B)** After greater and lesser wing of sphenoid was drilled out, frontal and temporal dura was exposed. Meningoorbital band (white arrow) was identified and cut. **(C)** Then, the orbital contents and temporal dura were more retracted medially and laterally, respectively. **(D)** After fine interdural dissection with temporal lobe retraction, the entire lateral wall of CS was exposed in which oculomotor nerve (black arrowhead), trochlear nerve (white arrowhead), trigeminal nerve and its branches are identified. F, frontal dura; T, temporal dura; PO, periorbita; V1, ophthalmic branch of trigeminal nerve; V2, maxillary branch of trigeminal nerve; V3, mandibular branch of trigeminal nerve.

**Figure 3:**
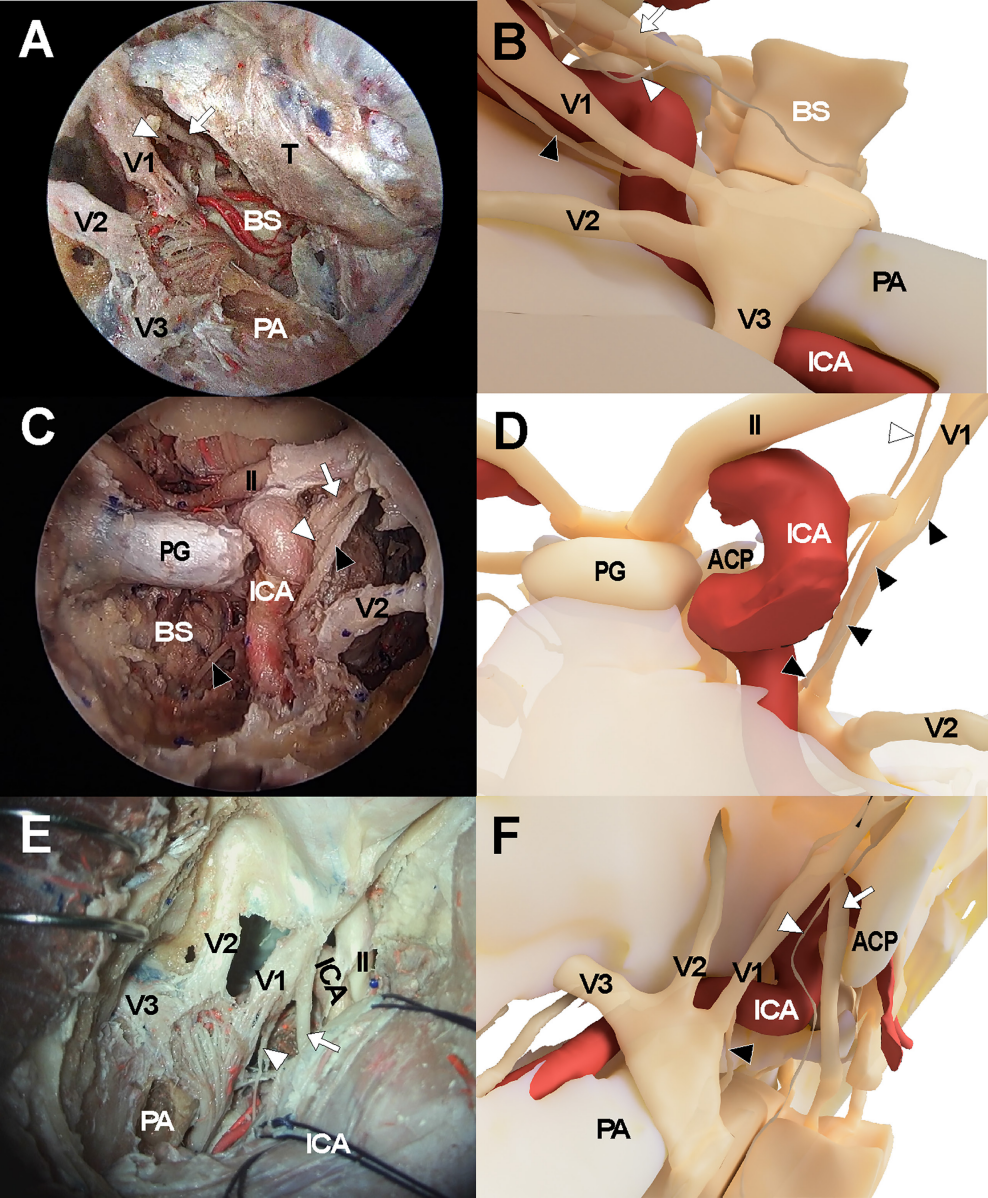
Cadaveric views and three-dimensional illustrations for cavernous sinus and surrounding neurovascular structures in the comparison with endoscopic transorbital approach **(A, B)**, endoscopic endonasal approach **(C, D)**, and transcranial approach **(E, F)**. The white arrows indicate oculomotor nerve while the white arrowheads and black arrowheads indicate trochlear nerve and abducens nerve, respectively. ACP, anterior clinoid process; ICA, internal carotid artery; II, optic nerve; PA, petrous apex; PG, pituitary gland; V1, ophthalmic branch of trigeminal nerve; V2, maxillary branch of trigeminal nerve; V3, mandibular branch of trigeminal nerve.

Dissection under ETOA was performed as previously described (4, 13, 14). A superior eyelid incision was made along the superior eyelid and was laterally extended (**Figure 2**). After making an incision on the orbicularis muscle and periosteum, the superolateral orbital rim was exposed. Subperiosteal dissection was extended towards the orbital apex while the orbital contents were medially retracted using a malleable retractor without violating the periorbital layer. The pyramid of sphenoid was drilled out. The meningolacrimal artery was identified and cut. After the meningoorbital band was fully exposed and cut, which facilitated medial retraction of the periorbita and lateral retraction of the temporal lobe dura, the greater and lesser wings of the sphenoid were further removed until full exposure of the superior orbital fissure (SOF) was achieved. The CS membrane consists of two dural layers; the outer layer dura propria continues with the frontotemporal dura and the inner layer, the true cavernous membrane, continues intraorbitally as the epineurium of the cranial nerves. Similar to the conventional transcranial CS approach, sharp and gentle interdural dissection was performed in the anterior-to-posterior direction to achieve wide exposure of the lateral CS wall (4, 15). Immediately after beginning the interdural dissection, ophthalmic (V1) and maxillary (V2) branches of the trigeminal nerve were identified at the front. After dissecting the space around these two large cranial nerve branches, the oculomotor and trochlear nerves were identified just above V1; the mandibular nerve (V3) and middle meningeal artery were identified just posterior to V2. After complete exposure of the lateral CS wall, the CS was explored though each surgical triangle and neurovascular structures inside the CS were identified (**Figures 3A, B** **)**.

### Endoscopic endonasal approach

The endonasal approach to the CS was performed in a standard manner using a two-surgeon binostril technique. The turbinates on the opposite side of the target CS were pushed to the lateral side. The middle turbinate, superior turbinate, and uncinate processes on the same side were removed. The posterior half of the nasal septum was removed, and the sphenoid rostrum and the ostia were removed. Then, the anterior wall of the sphenoid sinus and septum inside the sinus were removed to fully expose the sellar floor. Ipsilateral posterior ethmoidectomy was performed to enhance lateral access to the CS. Sellar floor bone was removed which extended to the level of the tuberculum sellae. The inferior compartment of the CS was accessed by removing the shell covering the carotid protuberance. The anterior and medial dural layer of the CS was removed to allow full exposure of intracavernous structures, including the ICA and cranial nerves (**Figures 3C, D** **)**.

### Microscopic transcranial approach

The frontotemporal craniotomy was performed in a conventional manner. After curvilinear scalp incision, the temporalis muscle was incised and retracted inferiorly with interfascial dissection until the zygomatic arch was fully exposed. The zygomatic arch was removed and a routine frontotemporal craniotomy was performed. The temporal skull base was fully exposed with dural retraction of the temporal lobe and was further flattened with drilling. The sphenoid lesser wing was removed using a high-speed drill until the meningoorbital band was identified. After the meningoorbital band was cut, the temporal dura was further retracted until the SOF entry point was exposed. The temporal dural layer was further dissected and separated from the temporal skull base circumferentially, and the foramen rotundum and foramen ovale were exposed after cutting the middle meningeal artery at the level of the foramen spinosum. During dissection, the greater superficial petrosal nerve was identified and preserved. Then, the dural layer covering the trigeminal nerve was cut and the outer layer of the CS was separated from the inner layer while preserving the cranial nerves within the lateral CS wall. The CS was then explored *via* the various surgical triangles demarcated by each cranial nerve (**Figures 3E, F** **)**.

We generated three-dimensional models of the CS that were based on the cadaveric dissection results of the three approaches to the CS. A three-dimensional model of anatomical structures including the skull, ICA, and cranial nerves, was reconstructed using Visible Korean data from Department of Anatomy, Ajou University School of Medicine. Using the three-dimensional model, the final views of the transorbital approach, transnasal approach, and transcranial approach were rendered. (**Figures 1**, **3B, D, F**).

## Results

**Figure 4:**
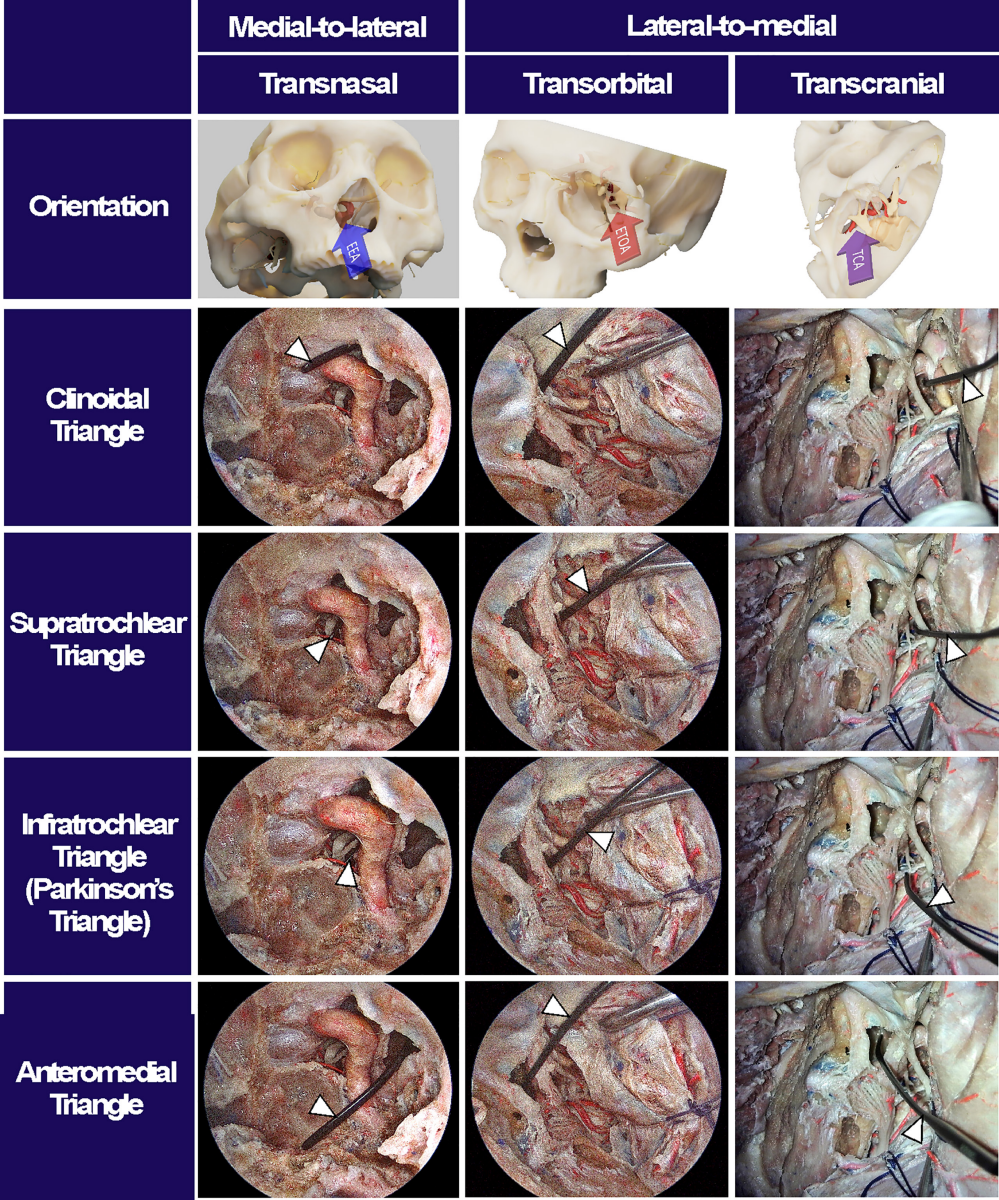
Surgical triangles of cavernous sinus with different approaches. Using a fresh cadaveric head, the surgical triangles to enter the cavernous sinus were simultaneously observed through an endoscopic endonasal approach (EEA), an endoscopic transorbital approach (ETOA), and a microscopic transcranial approach (TCA). The clinoidal triangle was made by removing the anterior clinoid process. The supratrochlear triangle consists of the oculomotor and trochlear nerves while the infratrochlear triangle, so called Parkinson’s triangle, was demarcated by trochlear nerve and ophthalmic branch of a trigeminal nerve. The anteromedial triangle indicated the space between the ophthalmic and maxillary branches of trigeminal nerve. After inserting an indicator (white arrowheads) into each triangle under TCA, the indicator’s location and angle were checked under EES and ETOA to enhance the understanding of surgical orientation in each approach.

Accessibility to the lateral compartment of the CS under the EEA was extremely limited mainly because of the tortuous ICA inside the CS. On the other hand, after the entire lateral wall of CS was exposed under ETOA, the oculomotor nerve, trochlear nerve, trigeminal nerve and its branches, which constitute the lateral wall of the CS, were easily identified and further dissected. The angle of attack under ETOA naturally aims towards the temporal lobe rather than frontal lobe, while providing a looking-down view. Thus, the viewing angle of the ETOA was very different from that of the EEA, which was obtained using a rather looking-up angle. The middle meningeal artery was seen lateral to V3. The anteromedial triangle demarcated by V1 and V2 was reached earlier during the ETOA, which provided a more spacious surgical window to enter the CS, compared with the surgical windows. As dissection was extended postero-superiorly, the oculomotor and trochlear nerves were identified above V1. Two surgical windows were identified and demarcated by the oculomotor nerve, trochlear nerve, and V1: the supratrochlear and infratrochlear triangles. The anterolateral triangle was also identified between V2 and V3, which did not provide a route into the CS. Using the three triangles, the lateral compartment of the CS was fully explored without damaging cranial nerves. The different trajectories towards the clinoid triangle, supratrochlear triangle, infratrochlear triangle, and anteromedial triangle were compared between the surgical approaches (**Figure 4**). It was difficult to visualize the cavernous segment of the ICA from outside the CS, which was covered by V1 during the ETOA. The abducens nerve was located between ICA and V1, running along medially to V1.

**Figure 5:**
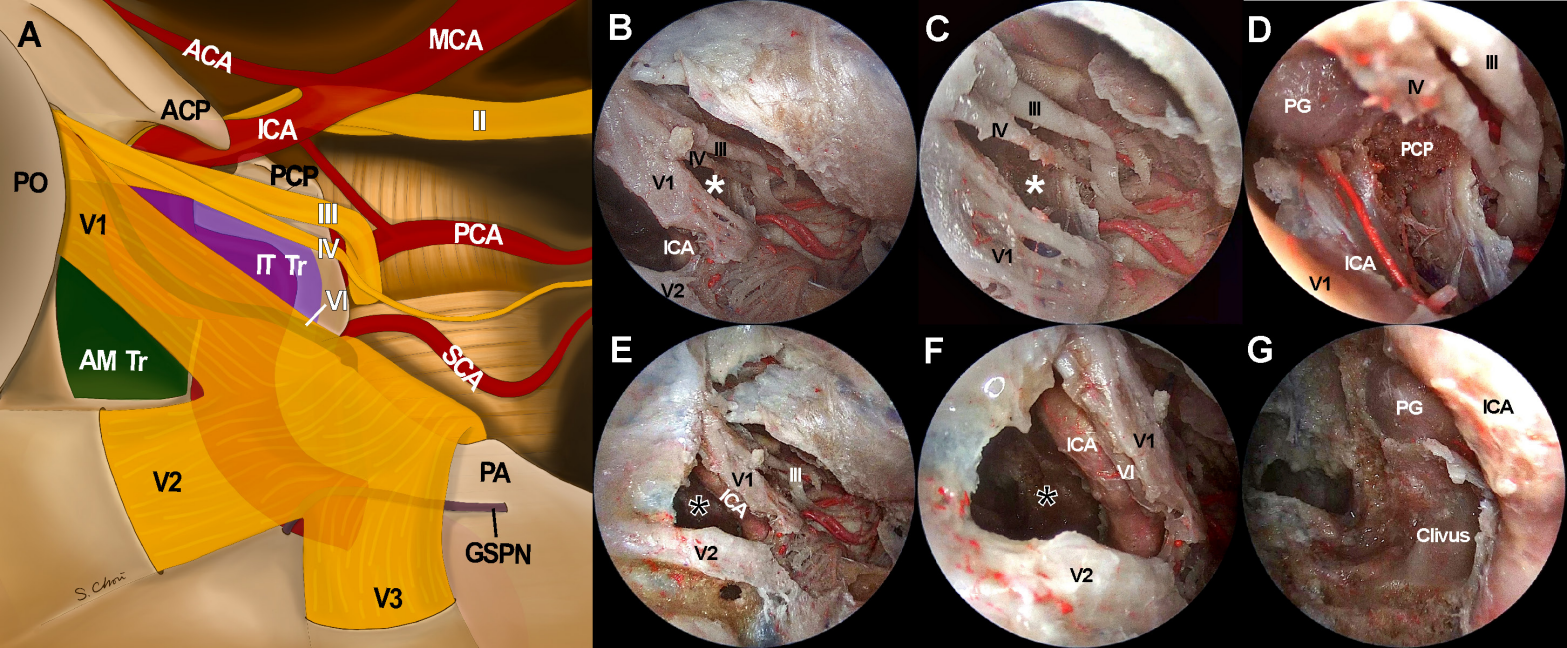
Access to intracavernous neurovascular structures through surgical windows under endoscopic transorbital approach (ETOA). **(A)** Illustration of skull base view under ETOA shows neurovascular structures inside and around cavernous sinus. Lateral orbital wall is removed and superior orbital fissure is opened. Sphenoid sinus also opened by drilling the sphenoid bone under the anteromedial triangle (green shadow). Infratrochlear triangle (purple shadow) was demarcated by the ophthalmic branch of a trigeminal nerve (V1) and a trochlear nerve. Dura including tentorium is widely excised. Abducens nerve and greater superficial petrosal nerve travel under the trigeminal nerve and V1 which are demonstrated transparently. **(B–D)** The infratrochlear triangle (white asterisks) allowed the access to the posterior region of cavernous sinus where the dorsal surface of internal carotid artery (ICA) was accessed. Posterior clinoid process was straightly directed by this window under an ETOA view. **(E–G)** The anteromedial triangle (black asterisks) was the main entrance to the lateral compartment of the cavernous sinus. The ventral surface of ICA horizontal segment was approached through this window. With a minimal retraction of V1 superiorly, abducens nerve was exposed just behind V1. Inferior compartment was also safely accessed through anteromedial triangle. ACA, anterior cerebral artery; AM Tr, anteromedial triangle; ICA, internal cerebral artery; II, optic nerve; III, oculomotor nerve; IT Tr, Inferior trochlear triangle; IV, trochlear nerve; GSPN, greater superficial petrosal nerve; MCA, middle cerebral artery; PCA, posterior cerebral artery; PCP, posterior clinoid process; PG, pituitary gland; PO, periorbita; SCA, superior cerebellar artery, V1, ophthalmic branch of trigeminal nerve; V2, maxillary branch of trigeminal nerve; V3, mandibular branch of trigeminal nerve.

When the endoscope tip was advanced through each surgical triangle into the CS, different CS regions with different neurovascular structures were visualized (**Figure 5**). The anteromedial triangle was the largest window where most of the lateral compartment was freely approached. The ICA and abducens nerve were also observed through the anteromedial triangle and just behind V1. Because the CS was accessed from the anterolateral side, it provided excellent accessibility to the trigeminal ganglion, the proximal part of cranial nerves, and even the tentorial incisura, whose accessibility was otherwise greatly limited under EEA. Compared with the TCA with a lateral-to-medial view, the ETOA visualized all these structures using less retraction of the temporal lobe. When the endoscope was directed inferiorly towards V2, the inferior compartment with the posterior ascending segment of the ICA was also visualized through the anteromedial triangle. The infratrochlear and supratrochlear triangles were much smaller windows. Using the ETOA, the approaching view through the supratrochlear and infratrochlear triangles was more directed towards the posterior part of the cavernous sinus, in which the posterior genu of the ICA and posterior clinoid process were visualized.

## Case illustration

**Figure 6:**
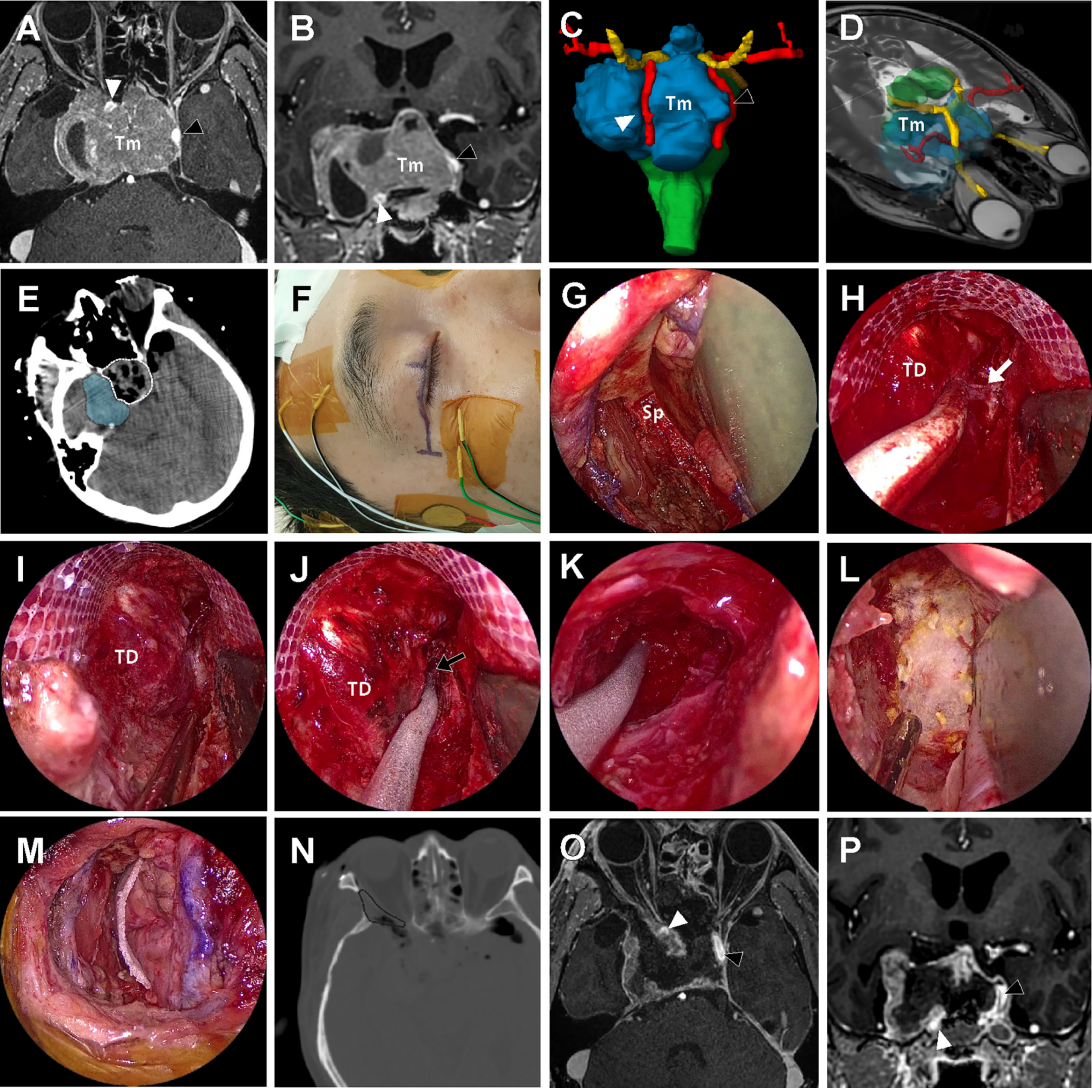
A 57-year-old male patient with history of repeated surgery and radiosurgery for an invasive pituitary adenoma. **(A, B)** Preoperative T1-weighted magnetic resonance imaging (MRI) with gadolinium enhancement shows a huge invasive pituitary adenoma (Tm) with right cavernous sinus (CS) invasion. The tumor pushed the right ICA (white arrow head) anteriorly and the left ICA (black arrow head) laterally. **(C, D)** On the three-dimensional volume rendering, the tumor (Tm, blue) was surrounded by optic apparatus (yellow) bilateral ICA (red). **(E)** Intraoperative computed tomography (CT) scan taken right after tumor removal under the endoscopic endonasal approach (EEA) revealed that the intrasellar tumor and suprasellar extension was successfully removed (white dotted line), but most of the tumor inside lateral compartment of CS remained (blue shadow). **(F)** About 3 cm-length incision along the upper eyelid crease was made for endoscopic transorbital approach (ETOA). The electrodes were placed to monitor the angle and direction of eyeball motion by triggered electrooculography and trigeminal sensory function by blink reflex. **(G)** After removal of lateral orbital rim, sphenoid bone (Sp) was exposed with medial retraction of orbital contents. **(H)** After extensive drilling of greater and lesser wing of sphenoid, the meningoorbital band (white arrow) was identified and cut to further separate the temporal dura (TD) and periorbita. **(I, J)** Interdural dissection was attempted but fine dissection was difficult because of postsurgical and postradiation fibrosis. Instead, window was made on the anteromedial triangle between V1 and V2. performed (black arrow). **(K)** As much tumor as possible in the lateral compartment of CS was removed through the anteromedial triangle. As tedious piecemeal removal of the hard tumor tissue was continued posteriorly until tumor removal cavity which was made under EEA was identified. **(L, M)** After meticulous bleeding control, dural and bony defect was reconstructed with synthetic surgical materials. **(N)** On the postoperative CT scan, the extent of bony removal for ETOA is indicated (black dotted line). **(O, P)** Subtotal removal of the tumor preserving bilateral ICAs (black and white arrow heads) and cranial nerves was demonstrated on the postoperative MRI.

A 57-year-old male patient presented visual discomfort and diplopia caused by a recurred pituitary adenoma. The tumor was previously treated three times using surgery and two times using gamma knife radiosurgery (GKS), until the tumor became large enough to cause neurological symptoms. Magnetic resonance imaging revealed a huge invasive pituitary adenoma (**Figure 6**). The optic apparatus was compressed by the suprasellar part of the tumor. The tumor extensively invaded the right CS and completely encased the ICA. Because the tumor in the lateral compartment of CS was too large to be treated by radiation alone and it was expected to adhere to neurovascular structures inside CS, the EEA alone was thought to be insufficient to access the tumor in the lateral compartment. It was decided to remove the tumor using a combined EEA and ETOA. Before surgery, we performed volume rendering of the tumor and the surrounding normal neurovascular structures using the Smartbrush^®^ of the Brainlab neuronavigation system (Brainlab AG, Munich, Germany), which greatly helped the surgeons understand the three-dimensional orientation of the target structure and thus simulated the surgical view under EEA and ETOA.

An extended transsphenoidal approach was performed in a standard fashion using a rigid endoscope 4 mm in diameter and 18 cm in length with 0° and 30° optic lenses (Karl Storz Endoscopy-America, Inc., Culver City, CA). Due to repeated previous surgical and radiosurgical treatments, the tumor was very hard and adhesive to surrounding structures and thus it was very difficult to identify clean dissection planes. Simple curettage was not effective to remove the hard tumor tissue, so it was forcefully removed in a piecemeal fashion using cup forceps. Consequently, it was very difficult and dangerous to explore the right side CS far laterally EEA. Especially, intracavernous ICA greatly limited accessibility to the lateral compartment of the CS under EEA.

After completion of the EEA, the extent and size of the remaining tumor was visualized using an intraoperative CT scan (AIRO mobile intraoperative CT; Brainlab AG, Munich, Germany). As majority of the tumor inside the right CS was not accessed by EEA and thus remained, the ETOA was then used. About a 3 cm-length eyelid incision was made with lateral extension, and the oculi muscle was incised and retracted. Using subperiosteal dissection, the periorbita was separated from the lateral orbital wall. The temporalis muscle was also dissected from the orbital lateral wall. Then, a piece of lateral orbital rim was removed creating a spacious window for the ETOA. Obviously, removal of the lateral orbital rim was not an essential procedure for ETOA, however, it provided greater surgical freedom with less orbital retraction. The right eyeball was covered with a corneal protector and the peritorbita was gently retracted medially. After the endoscope was introduced to the surgical field, the greater and lesser wings of the sphenoid were drilled out until sufficient exposure of the frontal and temporal dural surfaces was achieved. Because of the dense fibrosis caused by previous TCA and repeated GKS, clean interdural dissection was not possible. Instead, the anteromedial triangle demarcated by V1 and V2 was roughly localized and opened. The tumor inside the lateral compartment of CS was exposed and removed in a piecemeal fashion. Although a tumor-free margin was not obtained because of severe fibrosis, tumor tissue lateral to the ICA was more effectively explored under ETOA than the EEA, as previously demonstrated the cadaveric dissection. During dissection, the oculomotor and abducens nerves were localized using triggered electooculography (16). Sensory fibers of the trigeminal nerve were monitored by checking the blink reflex (17) and motor fibers (V3) were localized using electromyography. The ICA was frequently localized using Doppler ultrasonography during the tumor removal. Although small remnant tumors were left to avoid permanent damage of these critical neurovascular structures inside the CS, most of the tumor mass was successfully removed using the combination of the EEA and ETOA. The pathological examination revealed the diagnosis of a pituitary adenoma with 2% of the Ki-67 labeling index. Immunohistochemistry was positive only for SF-1 (steroidogenic factor 1) which was suggestive of a gonadotroph adenoma. The patient experienced transient right oculomotor palsy that resolved 3 months after surgery. After palliative re-radiation for the remnant tumor, patient has been followed up for 14 months without an evidence of further progression.

## Discussion

Surgical resection of CS tumors has been challenging for neurosurgeons. Because the CS is a complicated region surrounded and crowded by many neurovascular structures, various surgical approaches have been proposed; using them in combination is often required for large and extensive tumors (10, 18–21). For pituitary adenomas with extensive CS invasion, both transsphenoidal and transcranial surgery can be considered for tumor removal, and radiation and receptor-based pharmacotherapy are also a useful adjunctive treatment option for tumor control (19–23).

The ETOA is an emerging surgical corridor and provides access to various skull base regions. Trigeminal schwannomas, medial temporal lobe structures such as the amygdala and hippocampus, and sphenoid ridge meningiomas have been proposed as ideal targets of this novel approach (24–26). However, the CS has not attracted as much interest as other popular destinations of the ETOA. In this study, we conducted a cadaveric study to evaluate the feasibility of ETOA for access to the CS. Using previously reported interdural technique (4, 13), we found the CS lateral wall was effectively exposed under ETOA. Our study aimed not only to expose the lateral wall of the CS, but also to evaluate whether it was possible to access and explore the CS through the surgical triangles of the lateral wall.

Until the ETOA was proposed, TCA was the only surgical route used to explore the lateral compartment of the CS. Compared with the TCA, the ETOA has many advantages, such as a smaller skin incision, better cosmetic results, avoidance of temporal branch of the facial nerve palsy, no temporalis muscle atrophy, and less brain retraction (27–30). The approaching angle of the ETOA to the CS is quite different from the classical cavernous sinus approach used during the TCA, which has added a unique value to surgical routes to the CS. Because the ETOA aims more posteriorly along most of the cranial nerves, the Gasserian ganglion, the proximal part of the cranial nerves near the brain stem, and even the tentorial incisura were approached more easily. However, a narrow surgical corridor is a major limitation of the ETOA. Unlike the EEA where surgeons use two nostrils as external entry points providing a spacious surgical corridor for the endoscope and instruments, the ETOA generally allows only one external entry point. Therefore, during the ETOA, surgical freedom is profoundly limited when the retractor and endoscopic instruments are inserted altogether although several solutions are suggested to overcome this limitation (31, 32). Because the ETOA is a basically lateral-to-medial approach, a certain degree of cranial nerve manipulation in the lateral CS wall is inevitable. In addition, whereas the ICA is identified during the earlier phase of surgery under EEA, most of the cavernous ICA is concealed behind V1 which may impede identification of the ICA.

The CS is the region frequently invaded by pituitary adenoma. Although radiation is very effective for tumor control during pituitary adenoma management (21), CS exploration is often required for patients with large tumors inside the CS, patients with endocrine-active tumors such as acromegaly and Cushing’s disease, and patients with tumors refractory to radiation or pharmacotherapy. Because tumor consistency is often very soft and friable, surgical removal is more feasible with less chance of morbidity than other tumors such as meningiomas, chordomas, and nasopharyngeal malignancies. Most tumors medial to the ICA can be reliably approached and removed under EEA. However, it is not uncommon that tumors completely encase the ICA and certain parts of the CS cannot be visualized and accessed using the EEA. Especially when the tumor is very solid and thus not easily removed by simple curettage, a direct lateral-to-medial approach to hidden CS regions is necessary. Fernandez-Miranda et al. divided the CS into four compartments based on spatial relationships (i.e., superior, posterior, inferior, and lateral) (33). Because the EEA results in a view from the inferomedial side of the CS, it is difficult to reach the superior compartment and the lateral compartment blocked by the cavernous ICA. In contrast, when it is accessed *via* the supratrochlear, infratrochlear, and anteromedial triangles under ETOA, the lateral CS compartment located on the lateral side of the ICA can be easily reached. When it is directed more posteriorly through the supratrochlear and infratrochlear triangles, the superior CS compartment located above the horizontal ICA is directly approached. For the cases with tumor invasion into the clivus and tentorium, the supratrochlear and infratrochlear triangles provide a unique and valuable surgical route to these regions.

It is obvious that ETOA is now adopted to access various surgical regions, however, the advantages and risks should be individually discussed. Under the belief that each surgical region should be differentiated, Di Somma et al. recently proposed a staging system that categorizes various surgical regions based on the levels of difficulty in the ETOA. According to this scheme, the CS approach is a Stage 4 procedure and is considered to be one of the most technically demanding (34). Although the ETOA is obviously advantageous to provide a unique surgical route to the CS with a different angle of attack visualizing different CS regions distinguished from the EEA and TCA, it should not be adopted as the first line surgical corridor to the lateral compartment of CS. Because all these surgical approaches are complementary, the three-dimensional orientations of anatomical structures using these three different approaches is critical to consider during preoperative planning. The three-dimensional model of the CS developed during this study helped to enhance the surgeon’s understanding and thus can be used to improve surgical outcomes in the management of various skull base tumors.

## Conclusions

A cadaveric study found that the lateral CS wall was reliably accessed using the ETOA. Most of the lateral compartment was effectively explored through the anteromedial triangle; the infratrochlear and supratrochlear triangles provided a reliable entry point to the posterior compartment of the CS. We believe the ETOA provides a unique and valuable surgical route to the CS, with promising synergy when used with EEA and TCA. Our early experience with a clinical case convinces us of the efficacy of the ETOA for surgical management of CS-invading skull base tumors.

## Data availability statement

The original contributions presented in the study are included in the article/supplementary material. Further inquiries can be directed to the corresponding author.

## Ethics statement

The studies involving human participants were reviewed and approved by The Cadaveric Study Committee of the Yonsei University of Medicine and the Institutional Review Board of Severance Hospital, Yonsei University College of Medicine. The patients/participants provided their written informed consent to participate in this study. Written informed consent was obtained from the individual(s) for the publication of any potentially identifiable images or data included in this article.

## Author contributions

Conceptualization, EK; Methodology, I-HJ, JY, and EK; Software, SC, TR, and EK; Validation, EK; Investigation, I-HJ, JY, SC, SL, JK, TR, JH, and EK; Resources, EK; Data Curation, I-HJ, JY, and EK; Writing – Original Draft Preparation, I-HJ and JY; Writing – Review & Editing, EK; Visualization, EK; Supervision, EK; Project Administration, EK; Funding Acquisition, EK. All authors contributed to the article and approved the submitted version.

## Funding

This work was supported by the Basic Science Research Program through the National Research Foundation (NRF) of Korea (NRF-2021R1F1A1051996) funded by the Korean Ministry of Science, ICT and Future Planning (EK) and a faculty research grant of Yonsei University College of Medicine (6-2020-0224) (EK).

## Acknowledgments

The authors deeply appreciate Mr. Jun Ho Kim and Mr. Jong Ho Bang in Surgical Anatomy Education Center of Yonsei University College of Medicine for their technical support.

## Conflict of interest

The authors declare that the research was conducted in the absence of any commercial or financial relationships that could be construed as a potential conflict of interest.

## Publisher’s note

All claims expressed in this article are solely those of the authors and do not necessarily represent those of their affiliated organizations, or those of the publisher, the editors and the reviewers. Any product that may be evaluated in this article, or claim that may be made by its manufacturer, is not guaranteed or endorsed by the publisher.
